# Supplementary material for: Integration of multiple electronic components on a microfibre towards an emerging electronic textile platform
Source: Nat Commun. 2022 Jun 8;13:3173. doi: 10.1038/s41467-022-30894-4 (PMC9178034; doi:10.1038/s41467-022-30894-4)
Supplement: Supplementary file 1 — Supplementary Information [file 41467_2022_30894_MOESM1_ESM.pdf]

Supplementary Information

Integration of multiple electronic components on a microfibre towards emerging electronic textile platform

Sunbin Hwang[1,†], Minji Kang[2,†,§], Aram Lee[1], Sukang Bae[1], Seoung-Ki Lee[3], Sang Hyun Lee[4], Takhee Lee[5], Gunuk Wang[6] and Tae-Wook Kim[2,]*

[1]Functional Composite Materials Research Center, Korea Institute of Science and Technology, Jeollabuk-do, 55324, Republic of Korea
[2]Department of Flexible and Printable Electronics, LANL-CBNU Engineering Institute-Korea, Jeonbuk National University, 567 Baekje-daero, Deokjin-gu, Jeonju 54896, Republic of Korea
[3]School of Materials Science and Engineering, Pusan National University, 2, Busandaehak-ro-63-beon-gil, Geumjeong-gu, Busan 46241, Republic of Korea
[4]School of Chemical Engineering, Chonnam National University, 77 Yongbong-ro, Buk-gu, Gwangju 61186, Republic of Korea
[5]Department of Physics and Astronomy, and Institute of Applied Physics, Seoul National University, Seoul, 08826, Republic of Korea
[6]KU-KIST Graduate School of Converging Science and Technology, Korea University, 145 Anam-ro, Seongbuk-gu, Seoul, 02841, Republic of Korea
[†]These authors contributed equally.
[§]Present address: Chemical Materials Solutions Center, Korea Research Institute of Chemical Technology, 141 Gajeong-ro, Yuseong-gu, Daejeon 34114, Republic of Korea
*Author to whom correspondence should be addressed. E-mail: twk@jbnu.ac.kr

This document contains the following sections:

- Supplementary Fig. S1. Preparation of metal electrode pattern on a microfibre
- Supplementary Fig. S2. Optical microscope and SEM images of various metal patterns on a microfibre
- Supplementary Fig. S3. The schematic illustrations and optical microscope images of the device fabrication procedure
- Supplementary Fig. S4. Analysis on Al_2O_3 dielectrics and IGZO thin film
- Supplementary Fig. S5. Photograph of the measurement setup
- Supplementary Fig. S6. Thermoelectrical characteristics of temperature sensor on a microfibre
- Supplementary Fig.S7. Electrical characteristics of the IGZO FETs on the microfibre under repeated bending cycles
- Supplementary Fig. S8. Maximum bending test of the electronic fibre and electrical characteristics of the IGZO TFT on the electronic fibre
- Supplementary Fig. S9. Integrated electronic fibre embedded in a fabric
- Supplementary Fig. S10. Schematic of reel-to-reel process for mass production of electronic fibre

Other Supplementary Movie for this manuscript includes the following:

- Supplementary Movie 1 (.mp4). Electronic textile sewed by a syringe needle.

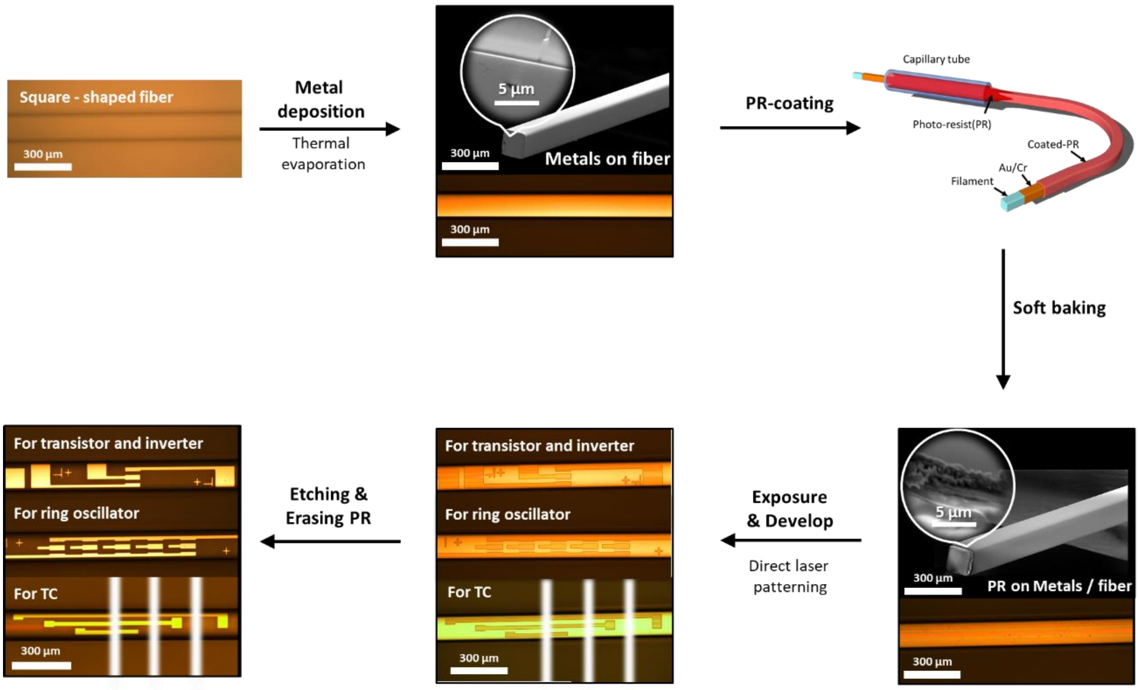

Fig. S1. Preparation of metal electrode pattern on a microfibre. Optical image showing the CTAC setup used for coating photoresist on a metal-deposited fibre substrate. SEM images showing an as-deposited PR film with a zoomed-in SEM image.

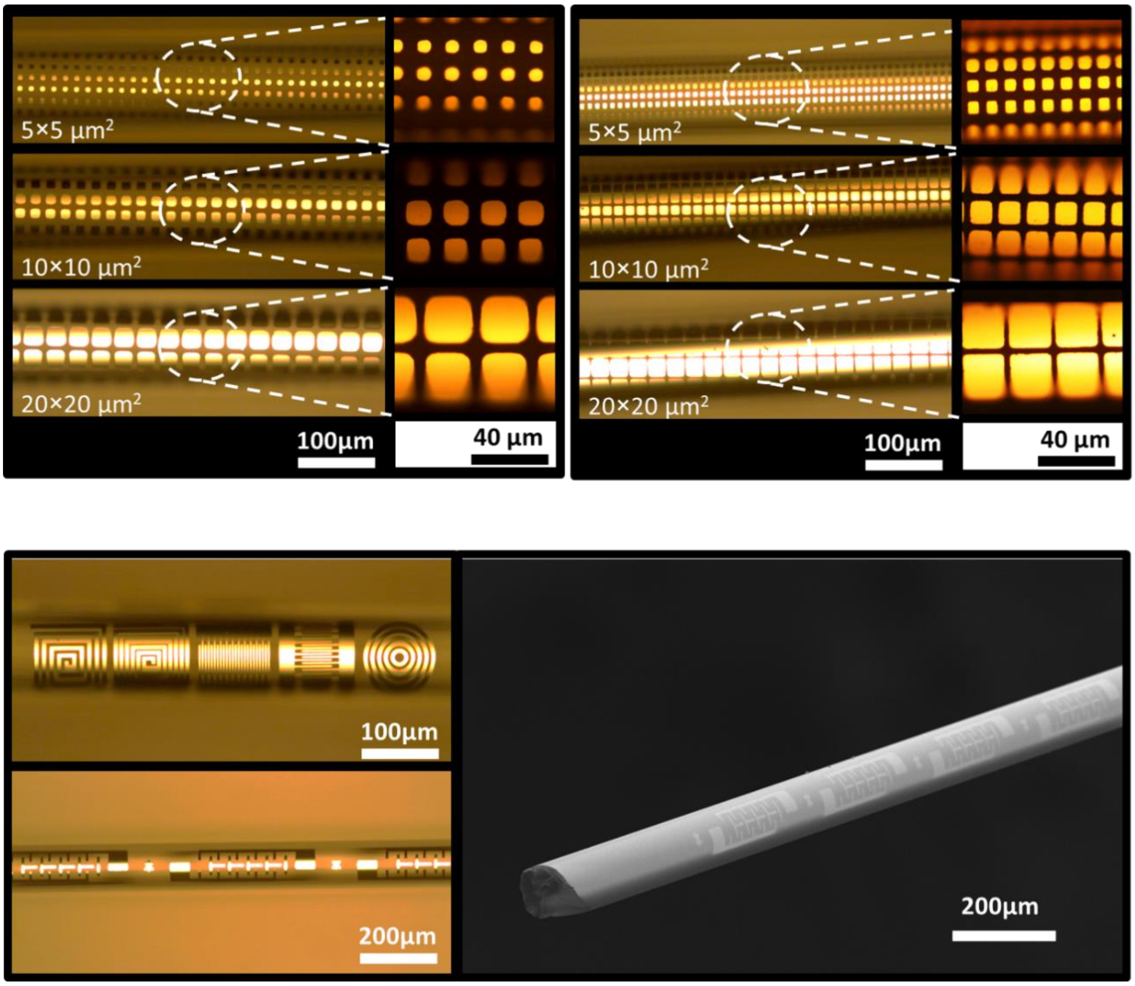

Fig. S2. Optical microscope and SEM images of various metal patterns on a microfibre. Top: The optical microscope images of square electrode pattern arrays having various sizes (5×5, 10×10, 20×20 μm^2) and pitches fabricated on cylindrical glass fibre ($\varphi = 125$ µm). Bottom: The optical microscope and SEM images of various patterned metal electrodes on cylindrical glass fibre.

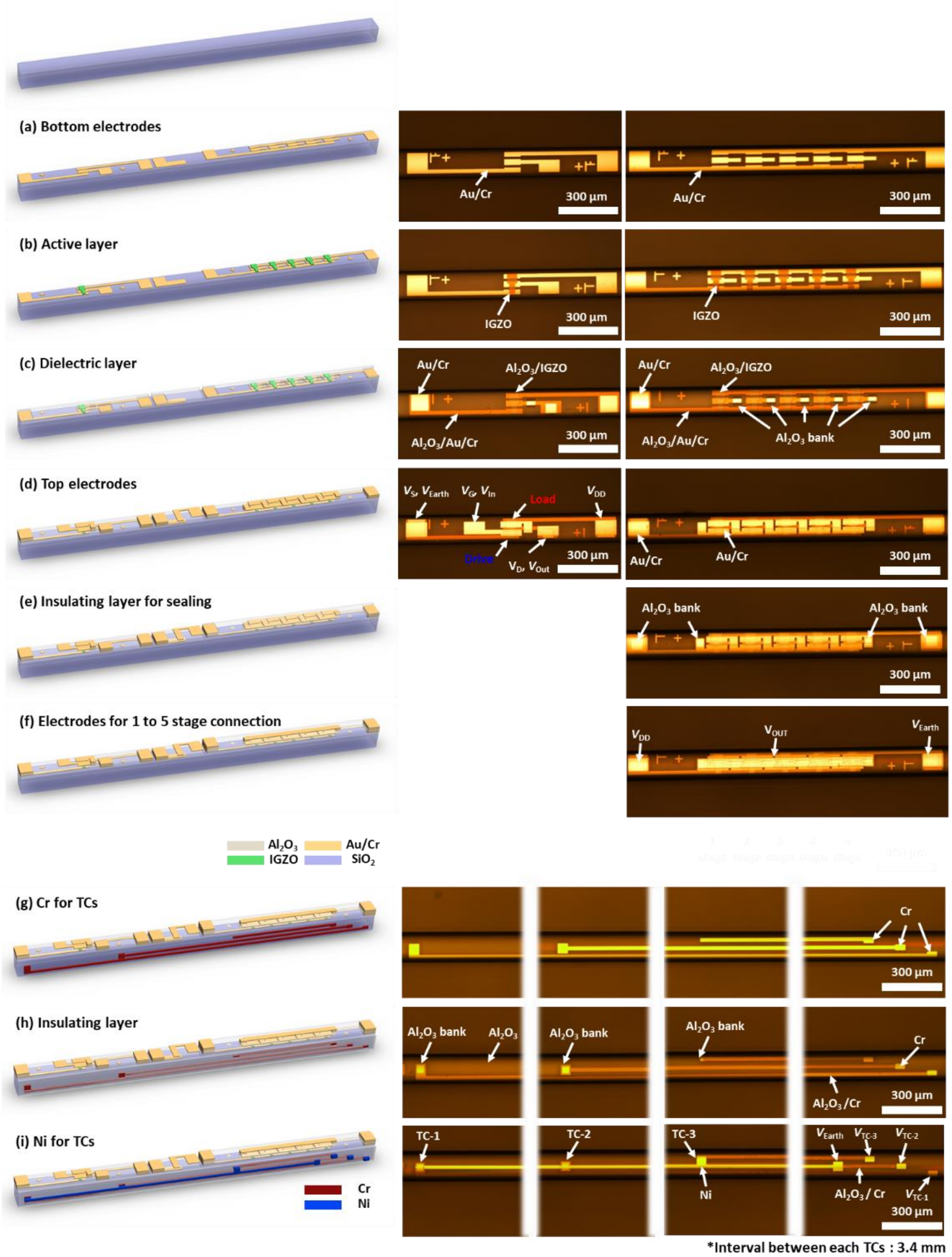

Fig. S3. The schematic illustrations and optical microscope images of the device fabrication procedure. (a) Gold/chromium (Au/Cr) bottom electrodes. **(b)** IGZO active layer for transistor, inverter, and ring oscillator. **(c)** Al_2O_3 dielectric layer contained via holes. **(d)** Au/Cr top electrodes. **(e)** Al_2O_3 insulating layer contained via holes for a transistor, an inverter, and a ring-oscillator. **(f)** Au/Cr short electrode to connecting the 1st and 5th stages of inverters for ring oscillator. **(g)** Cr electrodes on the side of the fibre,

which are used as one of the thermocouple (TC) components. **(h)** Al_2O_3 insulating layer contained via holes on the side of the fibre for the TC column. **(i)** Nickel (Ni) electrodes on the side of fibre, which are used as one of the TC components. (The interval between each TCs consisting of the TC column as shown in illustrations is 3.4 mm, which is drawn in a reduced size for presentation with all other TCs.)

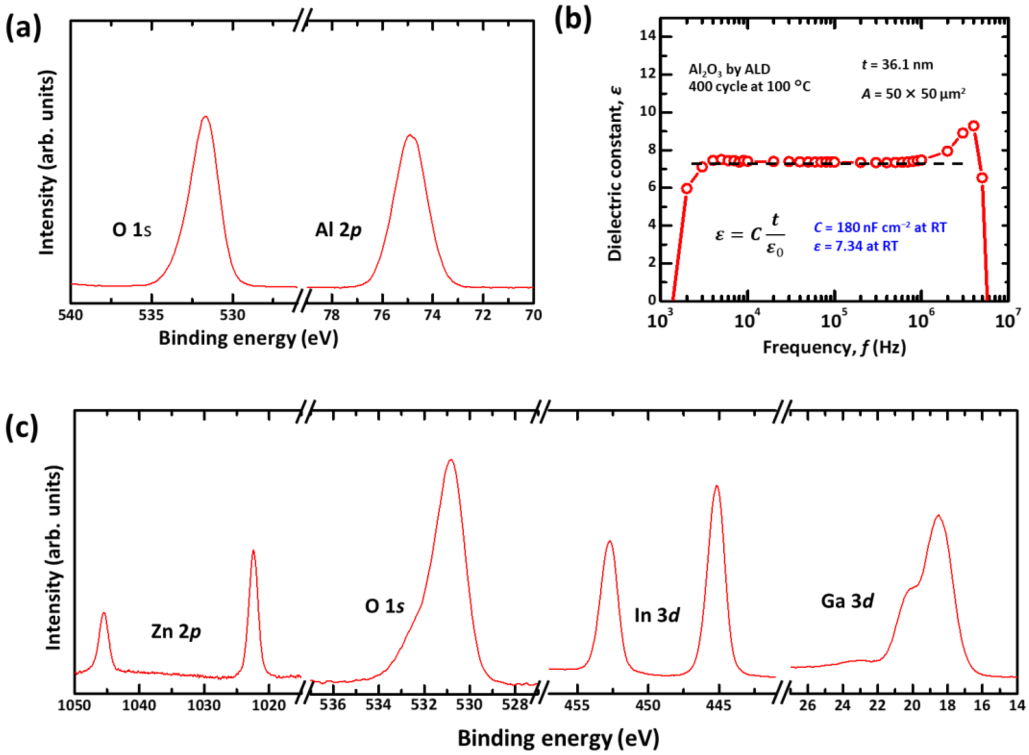

Fig. S4. Analysis on Al_2O_3 dielectrics and IGZO thin film. (a) X-ray photoelectron spectroscopy (XPS) narrow scan spectra of Al_2O_3 thin film fabricated by atomic layer deposition (ALD). **(b)** Dielectric constant (ε) versus frequency (***f***) characteristic of metal/dielectric layer/metal capacitor. **(c)** XPS spectra of IGZO thin film fabricated by a sputter. The inset tables in Fig. S4(a) and S4(c) show the peak binding energy, atomic percent, and the ratio of each atom compose each thin film.

The basic properties of the materials used in this study are described in Fig. S4. The chemical features of IGZO and Al_2O_3 thin films were identified by XPS. The relative quantifications of atomic percent (at%) were estimated from the major XPS peaks and analyzed using Avantage software (Thermo Fisher Scientific, Ltd.), which applies the XPS peak intensity taken by the area via the so-called first principles model and the elemental sensitivity factor methods. To estimate dielectric constant (ε) of Al_2O_3 thin film fabricated by atomic layer deposition (ALD), the capacitance measurement was performed using a semiconductor parameter analyzer (Keithley 4200 SCS). A capacitor composed of metal/dielectric/metal layers was fabricated on a square-shaped glass fibre, and the capacitance density ($\boldsymbol{C}$) was measured as a function of the frequency ($\boldsymbol{f}$). The $\boldsymbol{C}$ was converted to $\boldsymbol{\varepsilon}$ by using the below equation,

$$\boldsymbol{\varepsilon} = \boldsymbol{C}\frac{\boldsymbol{t}}{\boldsymbol{\varepsilon_0}},$$

Where $\boldsymbol{\varepsilon_0}$ is vacuum permittivity. The surface of a capacitor was 50 × 50 μm^2 and the thickness ($\boldsymbol{t}$) of Al_2O_3 thin film was 36.1 nm (ALD 400 cycle at 100 °C). Fig. S4(b) shows the $\boldsymbol{\varepsilon}$ versus $\boldsymbol{f}$ characteristics of the capacitors with the Cr/Au/Al_2O_3 /Au/Cr structure (30 nm of Au, 15 nm of Cr thickness for the bottom and top electrodes) and exhibited a

relatively high $\boldsymbol{\varepsilon}$ of 7.34 ($\boldsymbol{C}$ = 180 nF cm^{-2} at 10 kHz and room temperature), which is advantageous to lower the operating voltage of the device.[1]

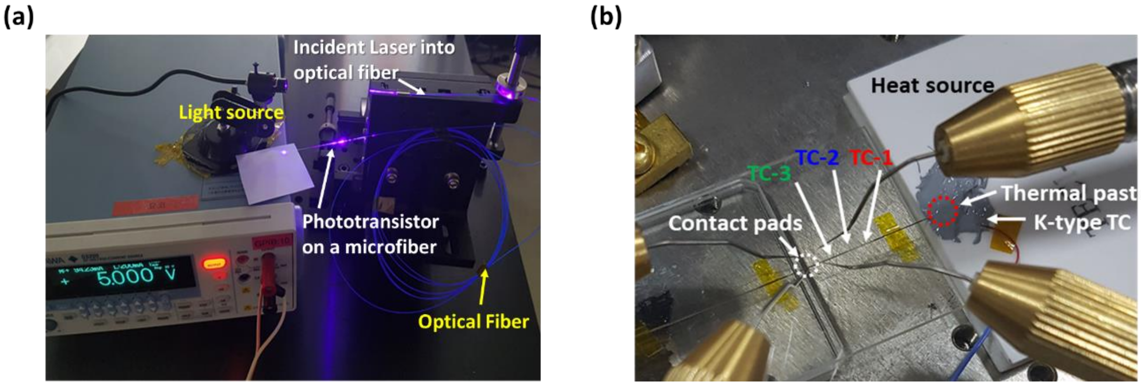

Fig. S5. Photographs of the measurement setup. (a) Optoelectrical measurement when the UV laser is irradiated through the fibre core and is propagated within the single FET fabricated on the optical glass fibre. **(b)** Thermoelectrical measurement during detecting thermal information.

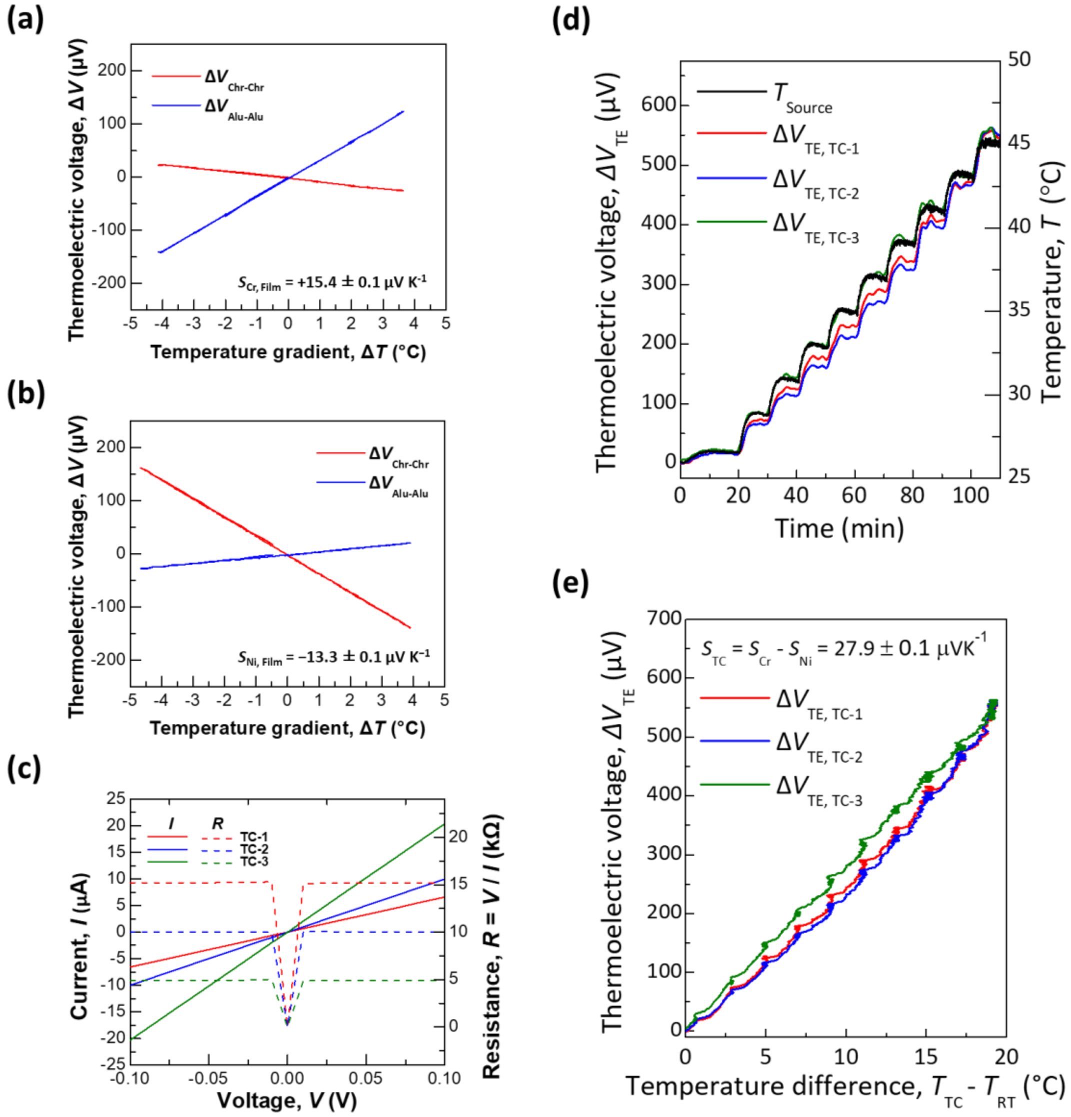

Fig. S6. Thermoelectrical characteristics of temperature sensor on a microfibre. Thermoelectric voltage (ΔV_{TE}) curves versus temperature gradient (ΔT) applied to **(a)** Chromium (Cr) and **(b)** Nickel (Ni) thin films. **(c)** Current (I)–voltage (V) curves and the calculated resistances of each thermocouple consisting of Cr and Ni thin film fabricated on the square-shaped glass fibre. **(d)** A plot of thermoelectric voltage versus time for the Cr–Al_2O_3–Ni thermocouples measured during a gradual increase in temperature. **(e)** Thermoelectric voltage as a function of temperature difference.

Seebeck coefficient (S) of chromium (Cr) and nickel (Ni) thin films were measured with a homemade setup as described in our previous reports.[2] To determine S, it is necessary to apply a temperature gradient (ΔT) across the sample and measure the thermoelectric voltage (ΔV_{TE}) and ΔT. Fine chromel and alumel wires (φ 50 μm, Nilaco) were used to resolve these problems. Chromel and alumel are alloys with known **S** and well-known as

materials for forming K-type thermocouples. When ΔT is applied to the sample, the temperatures at the hot and cold sides can be measured at the same time in addition to ΔV_{TE} generated from the sample. A typical ΔV_{TE} versus ΔT curve when measuring Cr and Ni thin films (3 mm of width, 23 mm of length, 30 nm of thickness) are shown in Fig. S6(a) and S6(b). From the blow equation, S_χ can be evaluated using either the chromel-chromel or alumel-alumel wire pair or both. Here, $S_{\chi,\text{ Chr-Chr}}$ and $S_{\chi,\text{ Alu-Alu}}$ are the S_χ estimated through the inner two chromel-chromel and alumel-alumel wire pairs, respectively.

$$\boldsymbol{S}_{\chi,\text{Chr-Chr}} = \frac{\Delta V_{\text{Chr-Chr}}}{\Delta T} + \boldsymbol{S}_{\text{Chr}},$$

$$\boldsymbol{S}_{\chi,\text{Alu-Alu}} = -\frac{\Delta V_{\text{Alu-Alu}}}{\Delta T} + \boldsymbol{S}_{\text{Alu}},$$

$$\boldsymbol{S}_\chi = \boldsymbol{S}_{\chi,\text{Chr-Chr}} = \boldsymbol{S}_{\chi,\text{Alu-Alu}} = \frac{\Delta V_{\text{Chr-Chr}} - \Delta V_{\text{Alu-Alu}}}{2\Delta T} + \frac{\boldsymbol{S}_{\text{Chr}} + \boldsymbol{S}_{\text{Alu}}}{2}$$

15.4 ± 0.1 μV K^{-1} and −13.3 ± 0.1 μV K^{-1} were obtained as **S** of Cr and Ni thin films ($\mathbf{S}_{\text{Cr, film}}$ and $\mathbf{S}_{\text{Ni, film}}$), respectively, from the linear slopes of the line fit to each $\Delta\mathbf{V}_{\mathbf{TE}}$−$\Delta\mathbf{T}$ curves and the equation, which matches well as **S** of thermocouple consisting of Ni and Cr ($\mathbf{S}_{\text{Cr, film}}$−$\mathbf{S}_{\text{Ni, film}}$) as discussed below. The **S** values of other reference samples were also measured to further confirm the accuracy of the measurement system. The previously reported **S** of chrome, gold, nickel, and platinum was 21.9 ± 0.2 μV K^{-1}, 2.0 ± 0.4 μV K^{-1}, −19.3 ± 0.4 μV K^{-1}, and −0.9 ± 0.65 μV K^{-1}, respectively. More detail about the schematic of the system, reference measurement, and theory can be found in previous reports.[2]

The quantity of heat flow (Q) across a temperature gradient can be expressed by Fourier's law as,

$$\boldsymbol{Q} = -\boldsymbol{A\kappa}\frac{\partial \boldsymbol{T}}{\partial \boldsymbol{L}} \qquad \text{(Equation 3: Fourier's law)},$$

where **A** is the cross-section area of the material, κ is the material's thermal conductivity, T is the temperature gradient as a function of the distance from a thermal source (L), as shown in Fig 4B. As discussed in Fig. S6(d) and S6(e), T at each TC junctions can be reversely calculated by using S of each TCs and ΔV_{TE} generated from each TCs, (See Equation 4).

$$\mathbf{T} = \frac{\Delta \mathbf{V}_{\mathbf{TE}}}{\mathbf{S}_{\mathbf{Cr}} - \mathbf{S}_{\mathbf{Ni}}} + \mathbf{T}_{\mathbf{RT}} \qquad \text{(Equation 4)}$$

T at each TCs as plotted in Fig. 4C was converted from ΔV_{TE} and Eq. 4 and showed a lower temperature in order away from the thermal source ($T_{Source} > T_{TC\text{-}1} > T_{TC\text{-}2} > T_{TC\text{-}3}$). The temperature distribution from the heat source at each sensor, as described in Fig. 4D, showed exponential decay due to heat loss from air convection instead of linear decay following Fourier's law. The temperature output of the thermoresistive TC column sensors fabricated on the 1D fibre indicates that it can be applied as a distributed temperature sensor.

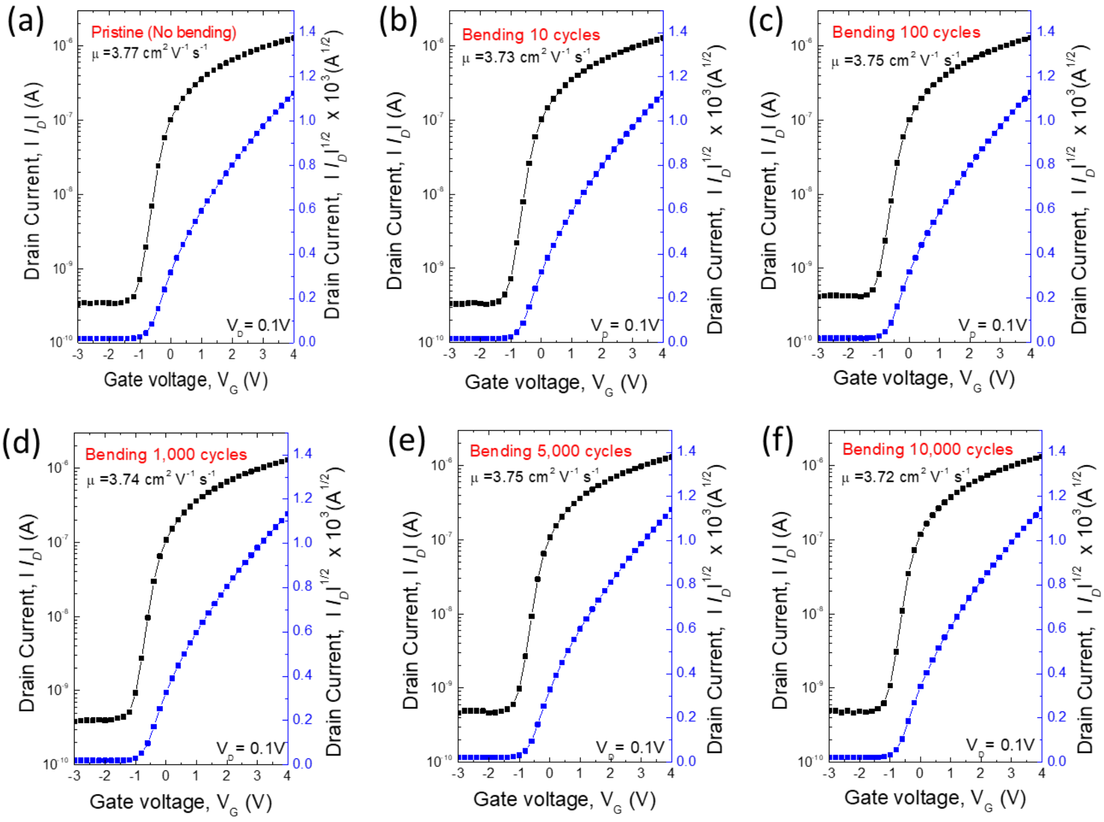

Fig. S7. Electrical characteristics of the IGZO FETs on the microfibre under repeated bending cycles. Transfer characteristics of the IGZO FETs on the microfibre during repeated bending up to 10,000 cycles at the fixed bending radius of 11.7 mm. **(a)** Pristine (No bending) and after bending **(b)** 10 cycles, **(c)** 100 cycles, **(d)** 1,000 cycles, **(e)** 5,000 cycles, **(f)** 10,000 cycles.

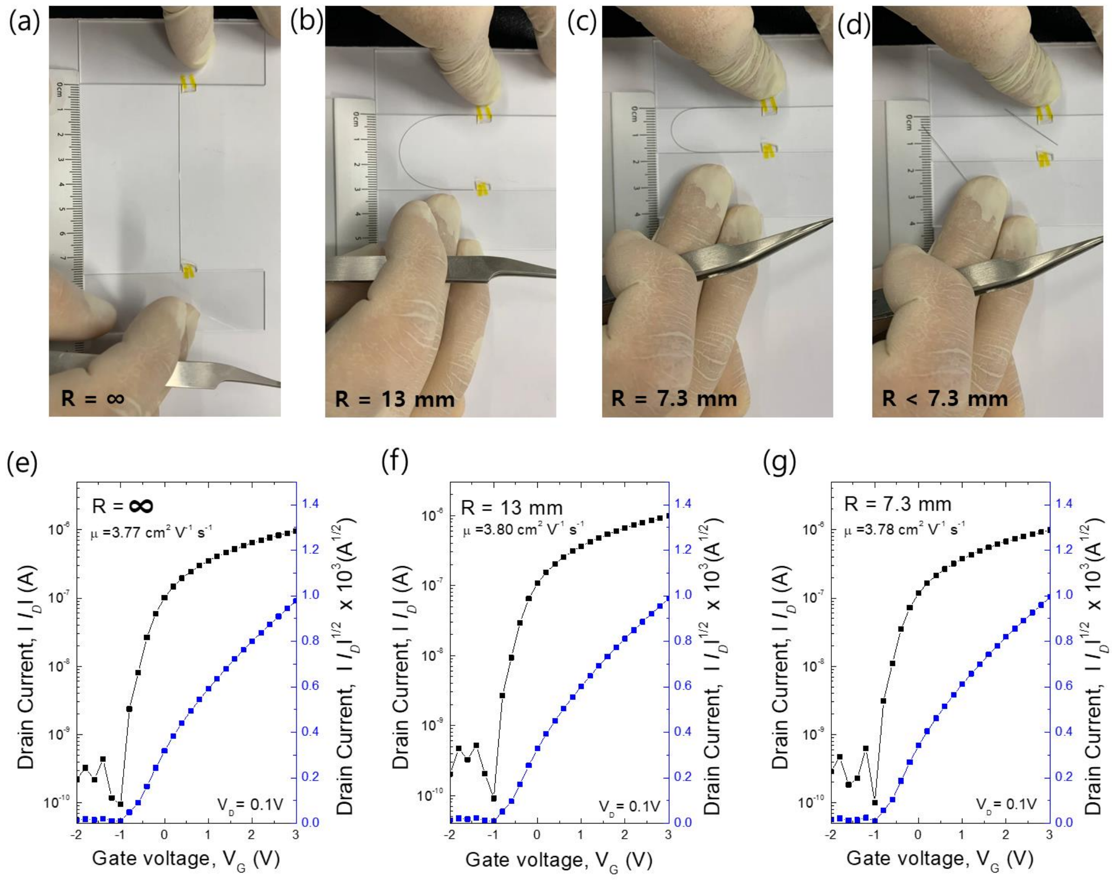

Fig. S8. Maximum bending test of the electronic fibre and electrical characteristics of the IGZO TFT on the electronic fibre. The bending radius (R) of **(a)** R = ∞, **(b)** R = 13 mm, **(c)** R = 7.3 mm, **(d)** R < 7.3 mm (Broken). The electronic fibre was broken at a bending radius of 7.3 mm. The calculated strain (ε) was 1.03 %. The electrical characteristics of the IGZO TFT on the electronic fibre at **(e)** R = ∞, **(f)** R = 13 mm, **(g)** R = 7.3 mm.

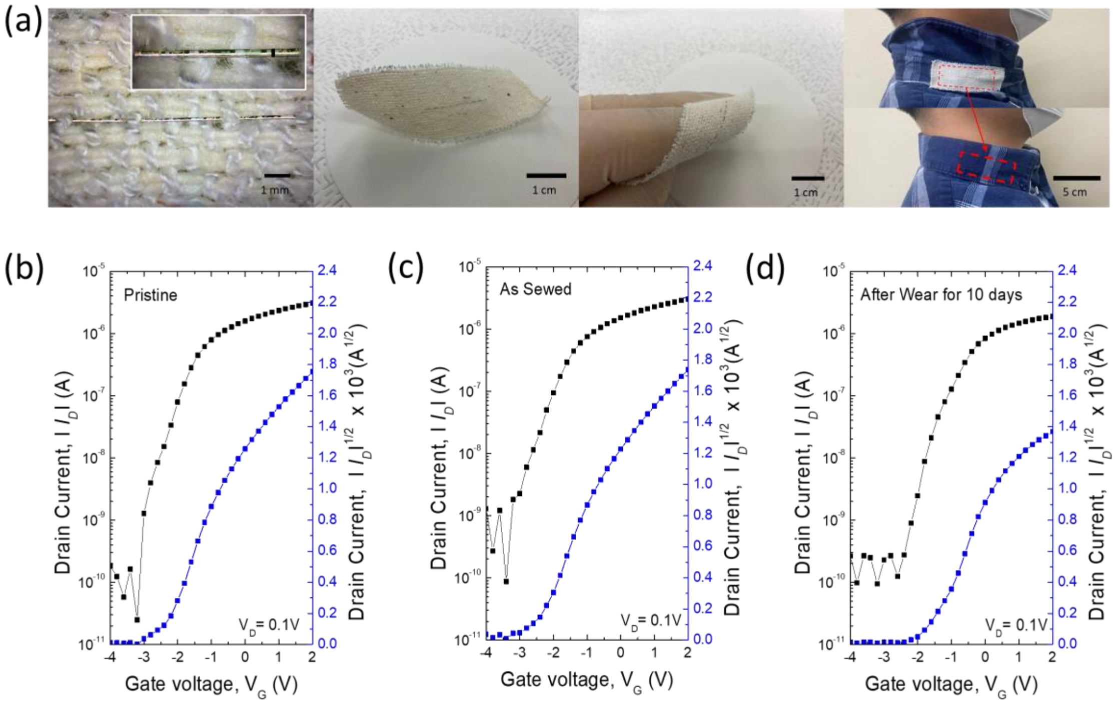

Fig. S9. Integrated electronic fibre embedded in a fabric. (a) Photographs of the electronic fibre sewn into fabric and inside the collar of a shirt. Transfer characteristics of the IGZO FETs on the fibre **(b)** at pristine, **(c)** as sewed, and **(d)** after wearing for 10 days.

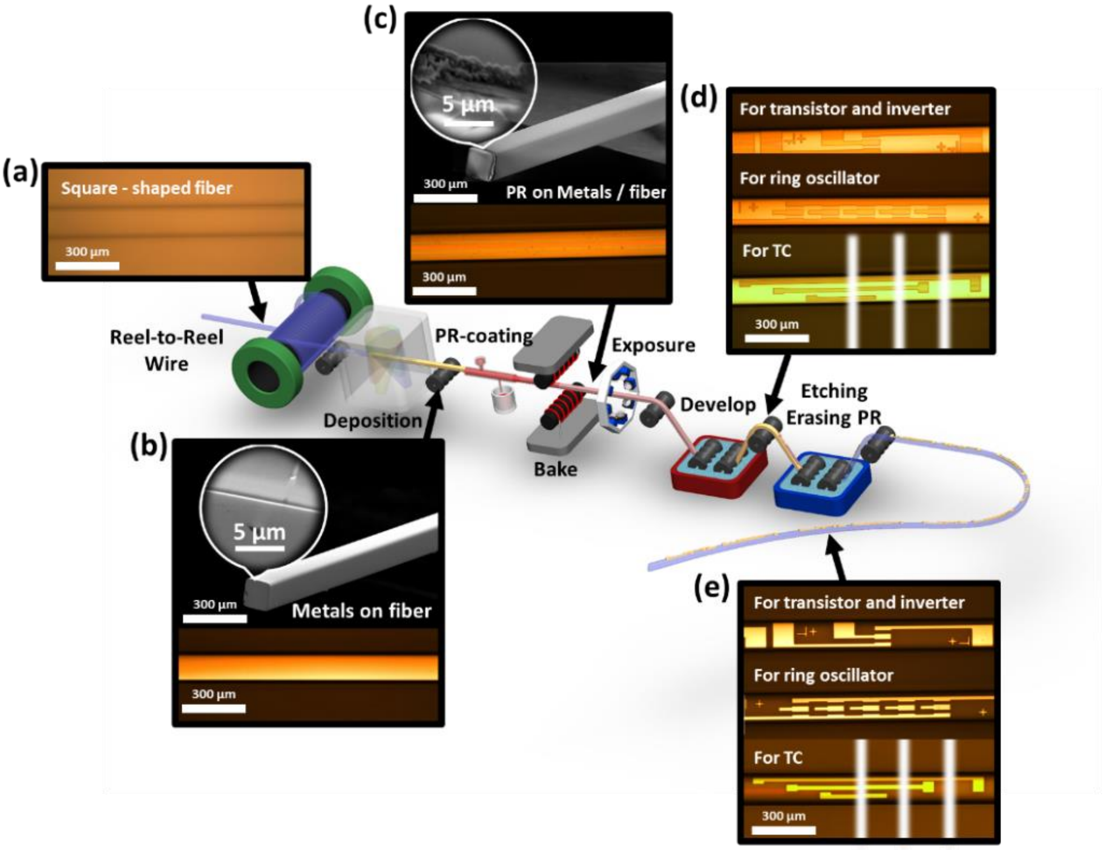

Fig. S10. **Schematic of reel-to-reel process for mass production of electronic fibre**. The photographs of electrode patterns formed on a monofilament during CTAC process, maskless photolithography, development, metal deposition, and wet etching process.

References

1. Wu, C. –H., Chang, K. –M., Huang, S. –H., Deng, I. –C., Wu, C. –J., Chiang, W. –H. & Chang, C. –C. Characteristics of IGZO TFT prepared by atmospheric pressure plasma jet using PE-ALD Al_2O_3 gate dielectric. *IEEE Electron Device Lett.* **33**, 552–554 (2012).
2. Hwang, S., Potscavage, W. J., Nakamichi, R. & Adachi, C. Processing and doping of thick polymer active layers for flexible organic thermoelectric modules. *Org. Electron.* **31**, 31–40 (2016).
