# Supplementary material for: Integration of multiple electronic components on a microfibre towards an emerging electronic textile platform
Source: Nat Commun. 2022 Jun 8;13:3173. doi: 10.1038/s41467-022-30894-4 (PMC9178034; doi:10.1038/s41467-022-30894-4)
Supplement: Supplementary file 3 — Description of Additional Supplementary Files [file 41467_2022_30894_MOESM3_ESM.pdf]

Description of Additional Supplementary Files

File Name: Supplementary Movie 1
Description: Electronic textile sewed by a syringe needle.
